# A new species of *Meryta* (Araliaceae) from the Marquesas Archipelago, French Polynesia

**DOI:** 10.3897/phytokeys.4.1408

**Published:** 2011-07-12

**Authors:** Frédéric Tronchet, Porter P. Lowry II

**Affiliations:** 1 Département Systématique et Évolution (UMR 7205), Muséum National d’Histoire Naturelle, CP 39, 57 rue Cuvier, 75213 Paris CEDEX 05, France; 2 Missouri Botanical Garden, P.O. Box 299, St. Louis, MO, 63166-0299, U.S.A

**Keywords:** *Meryta*, Araliaceae, French Polynesia, Marquesas, Hiva Oa, conservation

## Abstract

*Meryta pastoralis* F. Tronchet & Lowry, a new species from the island of Hiva Oa in the Marquesas archipelago, is described and illustrated. It differs from other Polynesian members of the genus by its fully free ovaries, a feature shared with one other species found in the region, *Meryta choristantha* (native to the Austral Islands), distinguished by its ovate to spatulate (vs. elliptic to obovate) leaf shape. A preliminary risk of extinction assessment indicates that *Meryta pastoralis* is Critically Endangered.

## Abstract (fr)

Une nouvelle espèce, *Meryta pastoralis* F. Tronchet & Lowry, de l’île d’Hiva Oa dans l’archipel des Marquises, est ici décrite et illustrée. Elle diffère des autres espèces du genre en Polynésie par ses ovaires totalement libres. Ce caractère est partagé uniquement par une autre espèce de la région – *Meryta choristantha* (endémique de l’archipel des Australes) – ses feuilles à limbe ovale à spatulé (vs. elliptique à obovale). Une première estimation du statut de de risque d'extinction indique que l’espèce *Meryta pastoralis* est en danger critique.

## Introduction

The genus *Meryta* J.R. Forst. & G. Forst. comprises a total of 37 species, 10 of which remain to be described (Frodin and Govaerts 2003, Tronchet and al. 2005a, Tronchet and Lowry unpubl. data), including the one proposed here. This distinctive group of monocaulous to well branched trees and shrubs is unique within Araliaceae in exhibiting a combination of simple leaves and a dioecious sexual system (Lowry 1988, Tronchet and al. 2005). *Meryta* occurs primarily in the South Pacific, extending from New Zealand and New Caledonia in the west across to Henderson Island in the east, with a single member present north of the equator in the Caroline and Mariana Islands (Lowry 1993, Tronchet and al. 2005a). Without exception, each species is endemic to a single island or archipelago, and most are known from just one or a few populations. While they rarely comprise a significant component of the vegetation, the distinctive morphology of *Meryta* species makes them easy to recognize. A few taxa are cultivated, most notably *Meryta pauciflora* Hemsl. ex Cheesman, which is often listed in horticulture catalogues, and *Meryta sinclairii* (Hook. f.) Seem., widely grown in New Zealand and occasionally elsewhere.

A recent phylogenetic study based on molecular sequence data (Tronchet et al. 2005a) showed that *Meryta* is monophyletic, a finding that is consistent both with morphology and wood anatomy (Oskolski et al. 2007). Within Araliaceae, *Meryta* belongs to the *Polyscias-Pseudopanax* clade, as defined by Plunkett et al. (2004, 2005; see also Lowry et al. 2004), and within this clade it is most closely related to the SW Pacific genus *Plerandra*, which was recently expanded to include species from Melanesia long included in *Schefflera* (Lowry et al. in press). The molecular phylogeny of Tronchet et al. (2005a) also revealed two principal subclades within the genus, one comprising a majority of the species (including the type) and the other with two species, one each from New Zealand and Fiji.

As part of our taxonomic revision of *Meryta*, we describe here a new species endemic to the island of Hiva Oa in the Marquesas archipelago so that it can be included in the forthcoming *Vascular Flora of the Marquesas Islands* being prepared by D. H. Lorence and W. L. Wagner. This distinctive new entity, first collected by P.A. Schäfer in 1975, represents a significant range extension for the genus and a noteworthy addition to the flora of the Marquesas.

## Systematics

### Meryta pastoralis

F. Tronchet & Lowry sp. nov.

urn:lsid:ipni.org:names:77112742-1

http://species-id.net/wiki/Meryta_pastoralis

Figs 1 -2 

#### Latin.

*Haec species quoad inflorescentiae structuram ac ovaria omnino libera Merytae choristanthae Harms (ex Insulis Australibus) simillima, sed ab ea foliis anguste obovatis usque spathulatis (vs. late ellipticis usque subobovatis), carpellis 5 ad 11 (vs. 5 vel 6 tantum) atque fructu maturitate 7-9* × *7-10 (vs. 8-12* × *10-15) mm distinguitur; etiam quoad foliorum aspectum M. raiateensi J.W. Moore et aliquantum M. lanceolatae J.R. Forst. & G. Forst. similis, sed ab eis (atque adeo ab omnibus congeneris Societatis Insularum ac Tuamotu) ovariis omnino liberis distinguitur.*

#### Type.

**French Polynesia.** Marquesas Islands, Hiva-Oa, Hanamenu valley off Hanamenu trail, in *Metrosideros-Weinmannia-Dicranopteris linearis* wet forest, 09°47'50"S, 139°05'35"W, 908 m, 2 August 2005 (fr), S. Perlman 19767 (holotype: P [P00398408]!; isotypes, PAP!, PTBG!, US!).

#### Description.

Monocaulous to sparsely branched tree, dioecious, Chamberlain architecture (tending toward Leeuwenberg architecture (Hallé, 2004) when very old, fide P.A. Schäfer, 1975), 3–6(–10) m tall, without milky sap. Leaves simple, alternate, grouped at branch ends; petiole 1.5–2.5(–4) cm long, 2–3 mm in diam., base enlarged, slightly clasping, lenticels rarely present abaxially, without dark green transverse striations adaxially when fresh; ligule present adaxially at the base of the petiole, persistent, free portion triangular to widely triangular, 5–11(–17) mm long, margins entire, membranous, apex obtuse to acute; blade green, shiny adaxially when fresh, slightly lighter green abaxially, narrowly obovate to spatulate, 20.4–40 × 5.2–9.8 cm (l/w ratio 2.76–5.52), base symmetric to asymmetric, offset 2–5(–10) mm, mostly positively centered in adaxial view, attenuate, margin entire, undulate distally, minutely revolute, apex acute to obtuse, the extreme tip obtuse to mucronate, chartaceous to coriaceous, glabrous on both surfaces; venation brochidodromous, light green-yellow when fresh, with prominent arches lacking intramarginal veins; midvein strong and massive, straight, without evident abaxial thickenings; secondary veins (12–)17–22 pairs, diverging from the midvein by (53–)57–69° (in the widest part of the blade), the distal ones less divergent, weakly recurved near the midvein then strongly recurved toward the margin, joining with the next arch at an obtuse angle without forming a clear intramarginal vein, intersecondary veins present; tertiary veins evident, not reaching the margin, straight to anastomosing at various angles, sometimes forming convex arcs in the intercostals zone; higher order veins visible in dry material, forming a fine, dense reticulum; veinlets forming quadri- to multi-angular areoles 0.5–1.5 mm in size. Juvenile foliage similar to adult, blade with an obtuse apex. Male material unknown (only old inflorescences seen). Female inflorescence terminal, erect, a raceme of spikes, with 1 degree of branching, axes and peduncle light green, bracts brown-red, primary axis 15–20 cm long, 5–8 mm in diam. at the base when fresh, subtended by persistent or sometimes caducous triangular cataphylls 13–24 mm long, without lenticels, margins entire, mostly to almost entirely membranous, apex obtuse, bearing a dorsal, ± foliaceous apicule; secondary axes 8–13(–17), 6–12 cm long (shorter distally), straight or sometimes slightly curved upward in the distal portion, diverging 35–60° from the primary axis, each subtended by a caducous, triangular to narrowly triangular bract 7–20 mm long, without lenticels, margin denticulate, mostly membranous, apex acute to acuminate, sometimes apiculate; spikelets with 7–15(–27) flowers, the proximal one inserted 3–15 mm from the base of the secondary axis. Female flowers white when fresh, sessile; bractlets caducous or sometimes persistent, broadly triangular, 3–5 mm long, margin weakly dentate, membranous, brown-red, partially covered by the flower and later the fruit, apex acute to obtuse; petals 5–11, caducous in fruit, ovate, 1–2.5 mm long, weakly cucullate, recurved when flowers receptive, with a pronounced adaxial groove, apex acute; androeciuum present, isostemonous, filaments and anthers developed but pollen sacs empty, stamens with filaments 0.5–1 mm long, anthers 0.5 mm long; ovary inferior, (5–)7 or 8(–11)-carpellate, nectar disc epigynous, 2 mm diam., styles weakly differentiated, to 1 mm long, stigmas 1.5–3 mm long at receptivity, often strongly recurved to twisted. Fruit yellow-green when young, light purple at maturity, remains of petals and stamens brown, ovaries entirely free and distinct from one another; drupes globose to sub-globose, 7–9 × 7–10 mm, smooth and fleshy when fresh, ribbed when dry, the ribs corresponding to the 5–11 pyrenes.

#### Distribution.

Known only from the type locality in the Mt. Temetiu/Feani area at ca. 900 m elevation along the Hanamenu trail on the island of Hiva Oa (Fig. 3), the largest and highest in the Marquesas archipelago.

#### Ecology.

*Meryta pastoralis* occurs on slopes and along streams in low stature, humid montane *Metrosideros-Weinmannia* forest at and above 900 m elevation.

#### Etymology.

This species is named in honor of P.A. Schäfer, who was the first to collect it in 1975 while conducting botanical inventory work in the Marquesas, and who has contributed much to our knowledge of the flora of the archipelago. The name Schäfer means shepherd in Alsatian, which when translated into Latin provides the basis for our choice of the epithet *pastoralis*.

#### Conservation status.

*Meryta pastorali*s is known from a single population along a wind-swept ridge below the highest point on Hiva Oa. With an Area of Occupancy of <10 km2 and an Extent of Occurrence that is probably no larger, it meets the area requirements for Critically Endangered status under criteria B1 and B2 of the IUCN Red List Criteria (IUCN 2001). While the vegetation in the immediate area is still largely intact, increasing impacts from feral pigs, human disturbance and fire during the dry season (D. Lorence, pers. comm.) represent growing threats to *Meryta pastoralis*, prompting us to assign it a provisional threat status of Critically Endangered (CR B1ab(i,ii,iii,v) + 2ab(i,ii,iii,v)).

#### Other specimens examined.

**French Polynesia.** Marquesas Islands, Hiva Oa, trail toward Hanamenu; in gulch off trail, low forest, 3 August 1988 (fr). S. Perlman 10206 (AD!, BISH! [2 sheets], F!, K!, MO!, NY!, P [P00372510]!, PAP!, PTBG!, US!); Teakatu, valley on north side of Hanamenu trail heading down to Hanamenu past summit crest, between Teakatau and Tepuna, along stream bottom in *Metrosideros-Weinmannia-Crossostylis* montane wet forest, [09°47'29"S, 139°05'41"W], 933 m, 26 August 1995 (st), S. Perlman & J.-Y. Meyer 14894 (BISH!, MO!, PAP!, PTBG!, US!); Feani area, Tepuna, in gulch to north of Hanamenu trail, down west side of summit crest to 1075 meters elev. and drop into gulch, in *Metrosideros-Weinmannia* forest, 09°47'49"S, 139°05'12"W, 900 m, 29 January 2003 (old ♂fl), S. Perlman 18335 (BISH, P [P00398011]!, PAP, PTBG, US); Feani area, Tepuna, off Hanamenu trail on west side of summit crest, at 1075 meters elev. into gulch north side of trail, scattered plants in *Metrosideros-Weinmannia* forest, 09°47'49"S, 139°05'12"W, 29 January 2003 (♀fl), S. Perlman 18336 (PAP, PTBG, US); Montagnes NW du Temetiu, entre la haute vallée de Hanamenu et la crête du Temetiu Feani, haute vallée, forêt humide, [09°48'00"S, 139°05'30"W], 925 m, 23 October 1975 (♀fl), P.A. Schäfer 5922 (BISH!, K!, MO! [2 sheets], NTBG!, P [P00372508 & P00372509]!, US!).

#### Discussion.

Material of our new species most closely resembles specimens of *Meryta choristantha* Harms from the Austral Islands, with which it shares a similarly structured inflorescence and totally free ovaries, but differs in leaf shape (narrowly obovate to spatulate in *Meryta pastoralis* vs. widely elliptic to slightly obovate in *Meryta choristantha*), number of carpels (5–11 vs. 5 or 6), and fruit size at maturity (7–9 × 7–10 vs. 8–12 × 10–15 mm). The leaves of *Meryta pastoralis* are similar in appearance to those of *Meryta raiateensis* J.W. Moore, and to a lesser degree *Meryta lanceolata* J.R. Forst. & G. Forst., both from the Society Islands, but our new taxon differs from these entities, and indeed all other species in the Society Islands and on Tuamotu, by its totally free ovaries.

Results from ongoing molecular phylogenetic work (Tronchet et al. 2005a, 2005b, unpubl. data) suggest that *Meryta pastoralis* is part of a small clade now understood to comprise four species, also including *Meryta sinclairii* from New Zealand, *Meryta tenuifolia* A.C. Sm. from Fiji, and *Meryta choristantha*, which together correspond to *Meryta* sect. *Choristomeryta* Harms (Harms 1938). Material here assigned to *Meryta pastoralis* was referred to informally as ‘M. schaeferi’ in Tronchet et al. (2005b).

**Figure 1.**
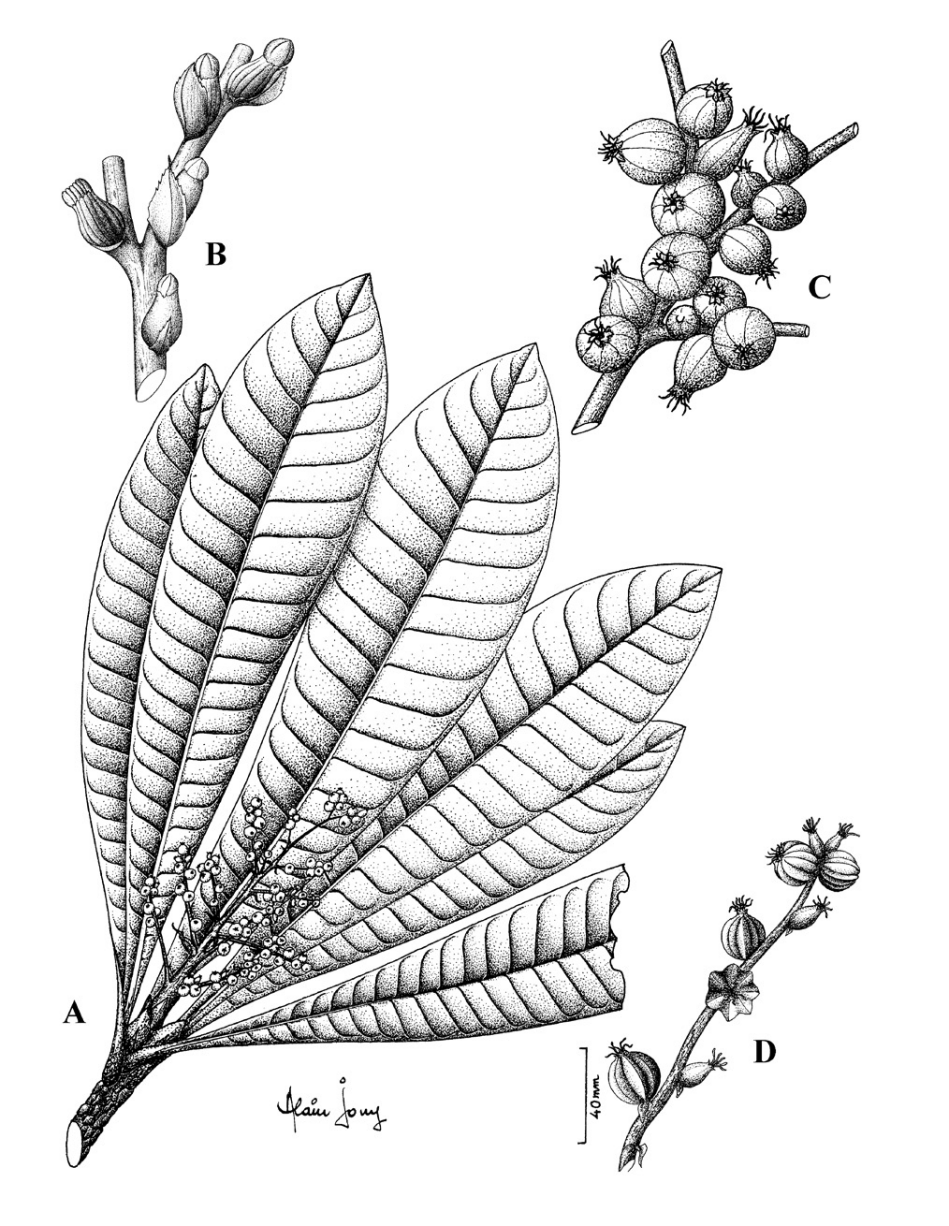
*Meryta pastoralis* F. Tronchet & Lowry. **A** branch with leaves and young infructescence **B** female flowers in bud subtended by bracts **C** female flowers at receptivity **D** young fruits. Line drawing by Alain Jouy from herbarium specimens; voucher Perlman 19767 (**A, D**), Schäfer 5922 (**B**), Perlman 10206 (**C**).

**Figure 2.**
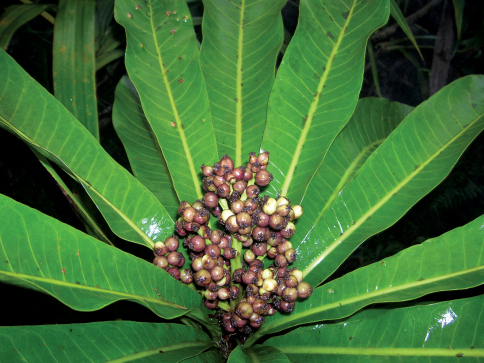
*Meryta pastoralis* F. Tronchet & Lowry. Photo of female plant in fruit (Photo by Steve Perlman of Perlman 19767).

**Figure 3.**
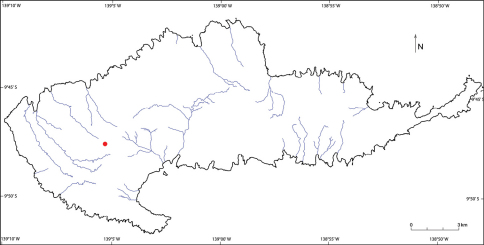
Distribution map of *Meryta pastoralis* F. Tronchet & Lowry on Hiva Oa Island in the Marquesas archipelago (blue lines indicate primary water courses).

## Supplementary Material

XML Treatment for Meryta pastoralis
